# Examining preharvest genetic and morphological factors contributing to lettuce (*Lactuca sativa* L.) shelf-life

**DOI:** 10.1038/s41598-024-55037-1

**Published:** 2024-03-19

**Authors:** Kathryn Chase, Catherine Belisle, Yogesh Ahlawat, Fahong Yu, Steven Sargent, Germán Sandoya, Kevin Begcy, Tie Liu

**Affiliations:** 1 Department of Environmental Horticulture, University of Florida, Gainesville, FL USA; 2 Department of Horticultural Sciences, University of Florida, Gainesville, FL USA; 3 Everglades Research and Education Center, University of Florida, Belle Glade, FL USA; 4 Bioinformatics, Interdisciplinary Center for Biotechnology Research, University of Florida, Gainesville, FL USA

**Keywords:** Senescence-associated genes, Shelf-life, Cultivars, Breeding, Postharvest, Pre-harvest, Plant sciences, Plant development, Stomata

## Abstract

Lettuce is a highly perishable horticultural crop with a relatively short shelf-life that limits its commercial value and contributes to food waste. Postharvest senescence varies with influences of both environmental and genetic factors. From a larger pool of romaine lettuce genotypes, we identified three genotypes with variable shelf lives and evaluated their leaf morphology characteristics and transcriptomic profiles at preharvest to predict postharvest quality. Breeding line 60184 had the shortest shelf-life (SSL), cultivar ‘Manatee’ had an intermediate shelf-life (ISL), and ‘Okeechobee’ had the longest shelf-life (LSL). We observed significantly larger leaf lamina thickness and higher stomatal index in the SSL genotypes relative to the LSL cultivar. To identify molecular indicators of shelf-life, we used a transcriptional approach between two of the contrasting genotypes, breeding line 60184 and cultivar ‘Okeechobee’ at preharvest. We identified 552 upregulated and 315 downregulated differentially expressed genes between the genotypes, from which 27% of them had an *Arabidopsis thaliana* ortholog previously characterized as senescence associated genes (SAGs). Notably, we identified several SAGs including several related to jasmonate ZIM-domain jasmonic acid signaling, chlorophyll *a*–*b* binding, and cell wall modification including pectate lyases and expansins. This study presented an innovative approach for identifying preharvest molecular factors linked to postharvest traits for prolonged shelf.

## Introduction

Despite lettuce (*Lactuca sativa* L.) popularity and nutritional value, the distribution, profits, and consumption of lettuce are limited by its relatively short shelf-life^1^. The main shelf-life-limiting characteristics affecting lettuce are wilting, yellowing of tissue, discoloration of cut edges and midribs from pinking and browning^1–3^. These traits of quality deterioration are undesirable for consumers and contribute to limited marketability and higher food waste.

Leaf senescence is a system of programmed cell death that consists of a series of complex, ordered processes responsible for breaking down cellular constituents into exportable nutrients, which are then transported to developing organs^4^. Postharvest senescence programs are dynamically mediated by senescence-associated genes (SAGs). These genes include those encoding receptors for abiotic and biotic stresses and developmental senescence cues, genes regulating secondary messengers such as Ca^2+^ and cyclic adenosine monophosphate (cAMP), genes involved in signal transduction pathways, and transcriptional regulators and their downstream genes that produce a physiological response^5^. General functional groups of SAGs include chlorophyll metabolism, hormone response and signal transduction, transcriptional regulation, protein degradation or modification, nucleic acid degradation, and lipid and carbohydrate metabolism^6^. Transcriptome analysis, forward genetic screening of mutant senescence phenotypes, and reverse genetics approaches have been employed over the last two decades to identify hundreds of SAGs and their common regulators^4^.

There is some evidence that postharvest phenotypes are likely associated with preharvest leaf traits. Analyses of quantitative trait loci (QTLs) for shelf-life, leaf biophysical properties, and leaf developmental traits identified QTLs for leaf cell wall biophysical properties of break strength, plasticity, and elasticity that correlated well with shelf-life^7^. These characteristics correlated with epidermal cell area, supporting the idea that postharvest shelf-life of leafy vegetables is linked to preharvest leaf traits and the small, strong cell-walled lettuce ideotype. Knock down of xyloglucan endotransglucosylase/hydrolase (*LsXTH*) gene expression using RNA interference (RNAi) produced cell wall loosening resulting in smaller leaves and leaf cells. Plants with reduced *LsXTH* expression had shelf-life extended by 6 days compared to the controls, suggesting that the higher surface area of cell wall associated with small cells contributed to extended shelf-life^8^. Additionally, an interspecific analysis of leaf physiological properties showed that long leaf lifespan correlated with high leaf mass per area. This trait represents a thicker leaf lamina or higher cellular density in the leaf tissue, or a combination of both^9^.

Molecular markers linked to postharvest traits for prolonged shelf-life are limited. Recently, studies focused on detached leaf senescence and young and old leaf lettuce leaves grown under various light source combinations have been used to identified molecular indicators of lettuce senescence^3,10^. Our study aimed to identify leaf traits and candidate genes as preharvest indicators of lettuce postharvest shelf-life. Markers such as these can serve as invaluable tools for lettuce breeding efforts. Identifying gene expression patterns during the preharvest period that correlate with shelf-life length could prove to be an important step in the development of markers to predict shelf-life phenotypes, which can be used to improve efforts to breed lettuce with improved postharvest shelf-life.

## Results

### Selecting lettuce genotypes based on visual characterization of shelf-life

**Figure 1:**
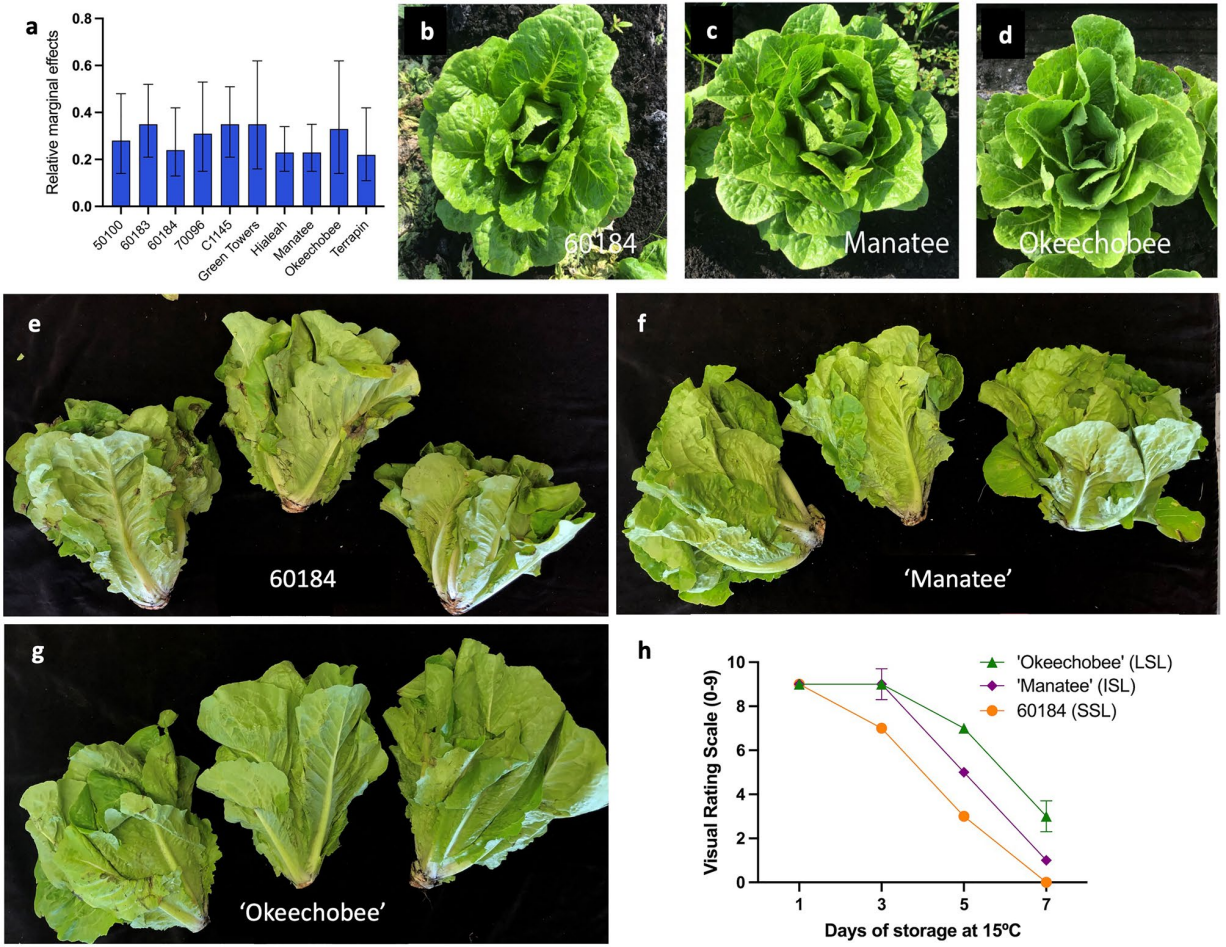
Head morphology and shelf-life variability of three romaine lettuce genotypes before and 5 days postharvest. (**a**) Relative Marginal Effects and their confidence intervals (95%) for visual ratings in 10 Romaine lettuce genotypes in the 2019–2020 season during storage for 5 days at 15 °C. (**b**–**g**) Head morphology of three lettuce genotypes at harvest (**b**–**d**), and after 5 days postharvest (**e**–**g**). (**b**,**e**) Experimental line 60184 (SSL), (**c**,**f**) ‘Manatee’ (ISL), and (**d**,**g**) ‘Okeechobee’ (LSL). (**h**) Median visual ratings of selected romaine lettuce genotypes during 7 days of storage at 15 °C. Bars indicate standard deviation.

To evaluate lettuce cultivars with variable shelf-life, 42 lettuce cultivars were previously surveyed based on visual ratings characteristics^11^. For this purpose, whole heads (*n* = 3) were visually evaluated for overall appearance using a hedonic rating scale adapted from Kader et al.^12^. Lettuce plants were rated as 1 if they were considered extremely poor quality and 9 if they were categorized as excellent quality. Ratings were based on the visible leaves, which included outer leaves and distal ends of middle and inner leaves. A lettuce head was considered good or marketable at an overall rating of at least 7 when there are no more than minor defects present on exposed leaves, while a head with a rating of fair or 5 has slight to moderate defects and was considered to be at the lower limit of marketability. We then selected 10 romaine germplasm having variable shelf lives (Fig. 1a) as indicated by their relative marginal effects analysis (Fig. 1a) of the visual ratings when lettuce was stored at 15 °C. We further selected three romaine lettuce germplasm with variable shelf-life for additional analyses. Experimental breeding line 60184 for short shelf-life (SSL), cultivars ‘Manatee’ as intermediate (ISL) and ‘Okeechobee’ for long shelf-life (LSL) (Fig. 1b–d). Significant differences were identified in the ANOVA-F analysis for the three genotypes further tested and the storage time. Interestingly, no significant differences were detected for the interaction genotypes × storage time. Our lettuce shelf-life rating assay showed that at 1 day after harvest, all genotypes had a median of 9 which is considered “excellent”; at 3 days after harvest, only 60184 deteriorated significantly faster with a rating of 7 compared with ‘Manatee’ and ‘Okeechobee’, which is still considered good shelf-life. The biggest deterioration rate occurred at 5 days after harvest when 60184 and ‘Manatee’ had a median of 5, indicating that the lettuce had some minor defects, while ‘Okeechobee’ maintained at 7 (Fig. 1e–g). By the last day of evaluation, at 7 days after harvest, only ‘Okeechobee’ had a value of 3 while 60184 had a value of 0 and ‘Manatee’ of 1 (Fig. 1h). Based on our lettuce shelf-life rating assay, we designated the experimental breeding line 60184 as the SSL genotype, ‘Manatee’ as the ISL cultivar, and ‘Okeechobee’ as the LSL cultivar.

### Leaf histological characteristics show little difference between lettuce genotypes

**Figure 2:**
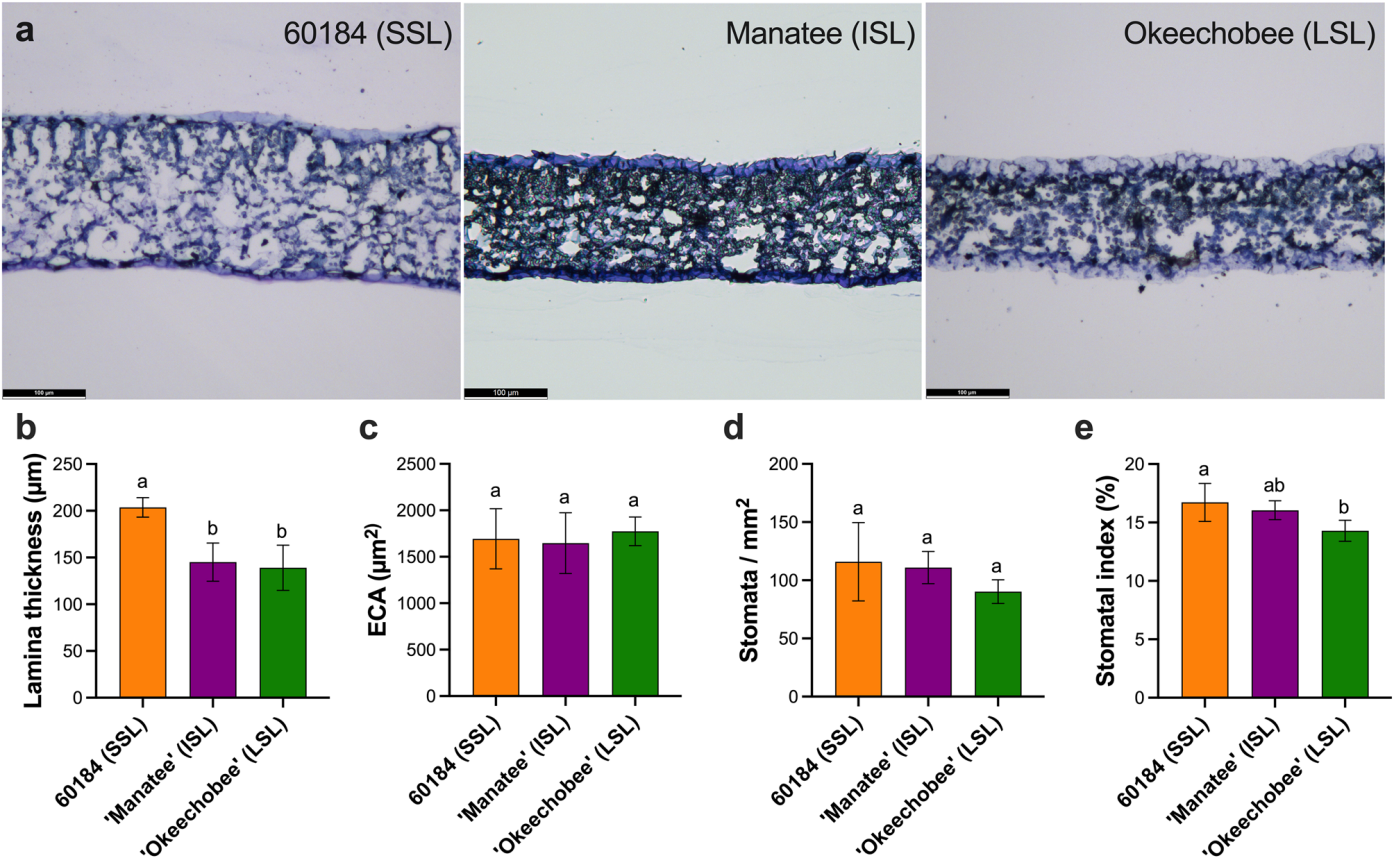
Lettuce leaf developmental characteristics. (**a**) Light microscope images of stained leaf lamina sections, taken at 10 × magnification. Scale bar = 100 µm. (**b**) Leaf lamina thickness (µm). (**c**) Epidermal cell area (µm^2^). (**d**) Stomatal density (stomata/mm^2^). (**e**) Stomatal index (%). Means with different letters indicate significant differences at *p* < 0.05; bars indicate standard deviation.

Pre-harvest leaf physical characteristics have been suggested as indicators of shelf-life as thicker leaves are favorable for processability and extended shelf-life^7,13^. Therefore, we characterized the leaf biophysical properties from the three selected genotypes (60184 (SSL), ‘Manatee’ (ISL) and ‘Okeechobee’ (LSL)) with variable shelf-life. We hypothesized that leaf thickness would increase across the representative genotypes analyzed for shelf-life. To test if these relationships between preharvest leaf traits and shelf-life were shown in our selected lettuce genotypes, we examined their leaf developmental characteristics, including leaf lamina thickness, ECA, stomatal density, and stomatal index (Fig. 2). Leaf lamina thickness was significantly larger in 60184 (SSL) relative to ‘Manatee’ (ISL) and ‘Okeechobee’ (LSL) (Fig. 2a,b). Epidermal cell area was not significantly different between genotypes (Fig. 2c). There was a decrease in stomatal density and stomatal index from SSL to LSL genotypes, but the only significant differences were found in stomatal index between 60184 (SSL) and ‘Okeechobee’ (LSL), with ‘Okeechobee’ (LSL), having a significantly lower stomatal index (Fig. 2d,e). Interestingly, among these romaine genotypes, variation in leaf developmental traits were not correlated with variation in shelf-life length. These data indicate that other factors may explain the variation. Therefore, we searched for genetic and molecular players that might explain the observed shelf-life variation among our genotypes.

### Identification of molecular markers associated with lettuce senescence

To understand the genetic variation of senescence and its contribution to shelf-life, we selected two lettuce genotypes with contrasting shelf-life and performed transcriptomic analyses. We used 60184 (SSL) and ‘Okeechobee’ (LSL) as plant material (Fig. 1a,c,e). An average of 33,103,823 reads across the six samples mapped uniquely to the *L. sativa* cv. Salinas reference genome. The percent alignment of mapped transcripts averaged 87.63% of transcripts with unique mapping, indicating adequate library preparation (Supplementary Table S1). Approximately 25,000 transcripts were mapped based on reads from each sample (Supplementary Table S1).

**Figure 3:**
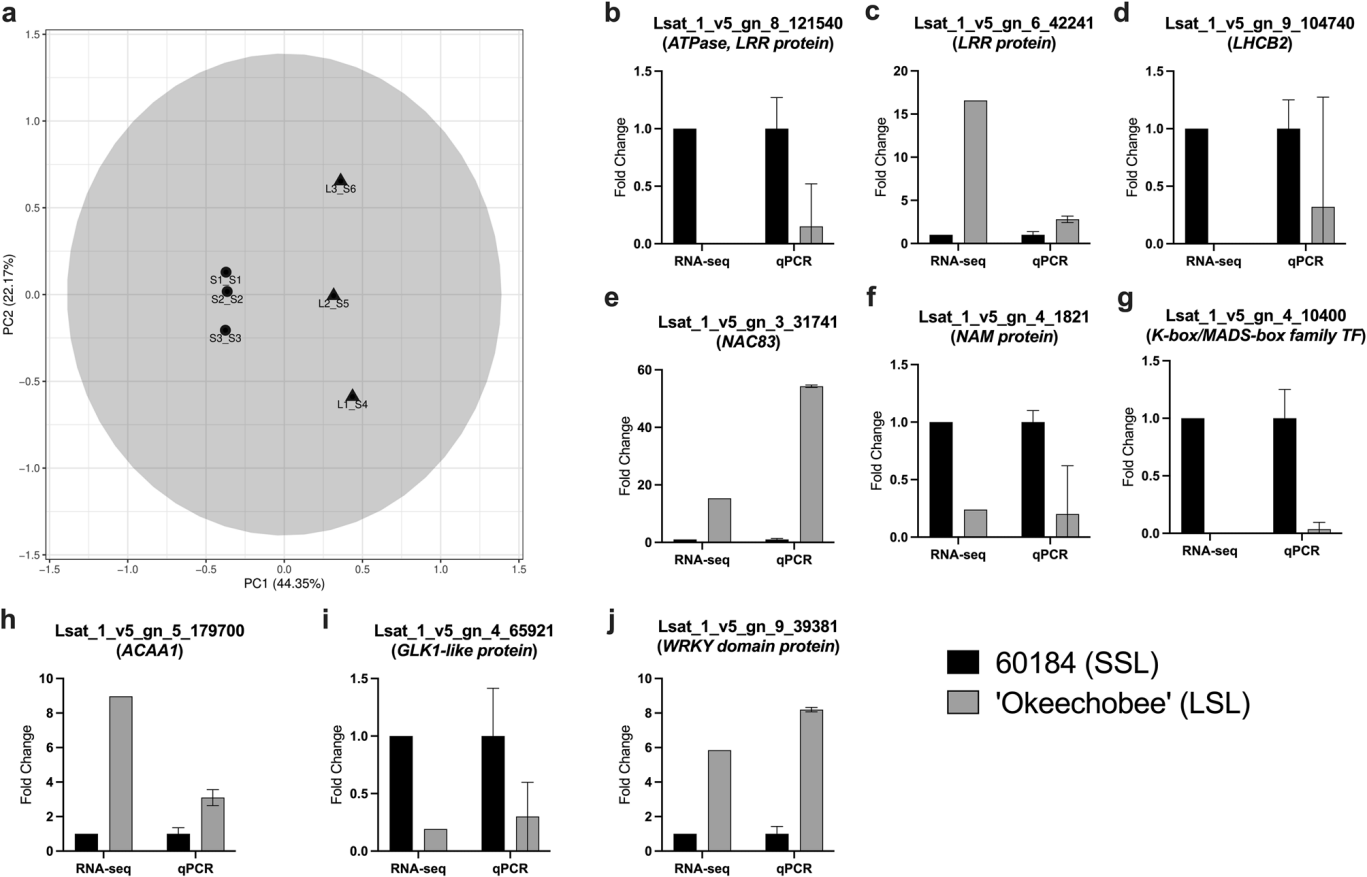
Transcriptional expression analysis of lettuce SAGs. (**a**) PCA plot of lettuce RNA-seq data, pairwise comparison between 60184 (SSL) and ‘Okeechobee’ (LSL). Circles correspond to 60184 samples and triangles correspond to ‘Okeechobee’ samples. (**b**–**j**) Expression of ‘Okeechobee’ (LSL) genes relative to 60184 (SSL) genes in RNA-seq and RT-qPCR assays, expressed as fold change.

To assess the replicability of the biological samples, a PCA was performed. The first two principal axes explained 44.35% (PC1) and 22.17% (PC2) of the variance (Fig. 3a). These axes represented genotype and biological replicates, respectively. The PCA for each pairwise comparison showed that genotype biological replicates cluster together, and there were no outliers. The tighter clustering of 60184 biological replicates along the PC2 axis shows there was less variation between individuals of this genotype than in the ‘Okeechobee’ biological replicates (Fig. 3a).

To validate the RNA-seq results, we used two criteria to select nine genes for expression analysis using RT-qPCR. Candidate genes were selected based on high fold change between SSL and LSL and their previous characterization as putative SAGs based sequence homology to characterized *A. thaliana* SAGs. Selected genes included an *ATPase* (Lsat_1_v5_gn_8_121540) and a light harvesting/*CAB* gene (*LHCB2*; Lsat_1_v5_gn_9_104740), as well as seven DE lettuce genes possessing an *A. thaliana* senescence-associated ortholog. These lettuce genes were comprised of two no apical meristem genes (*NAC83;* Lsat_v5_gn_3_31741 and *NAM;* Lsat_1_v5_gn_4_1821), a leucine-rich repeat protein (*LRR*; Lsat_1_v5_gn_6_42241), an acetyl-CoA acyltransferase (*ACAA1*; Lsat_1_v5_gn_5_179700), a MADS-box-type transcription factor (TF) (Lsat_1_v5_gn_4_10400), a Golden-like1-related TF (*GLK1*; Lsat_1_v5_gn_4_65921), and a *WRKY* TF (Lsat_1_v5_gn_9_39381) (Supplementary Table S4). For the nine genes, expression in ‘Okeechobee’ (LSL) exhibited similar trends relative to 60184 (SSL), as estimated by RNA-seq and RT-qPCR assays (Fig. 3b–j). For instance, *NAC83* (Lsat_1_v5_gn_3_31741) and the *WRKY* TF had increased expression in ‘Okeechobee’ (LSL) in both RNA-seq and RT-qPCR assays, with over 15-fold and 5-fold increases, respectively (Fig. 3e,j). The ATPase gene and MADS-box TF, which had the most significant decreases in expression in ‘Okeechobee’ (LSL) in the RNA-seq experiment, had similarly low fold changes in the RT-qPCR assay (Fig. 3b,g).

### Functional enrichments of DE genes during preharvest

**Figure 4:**
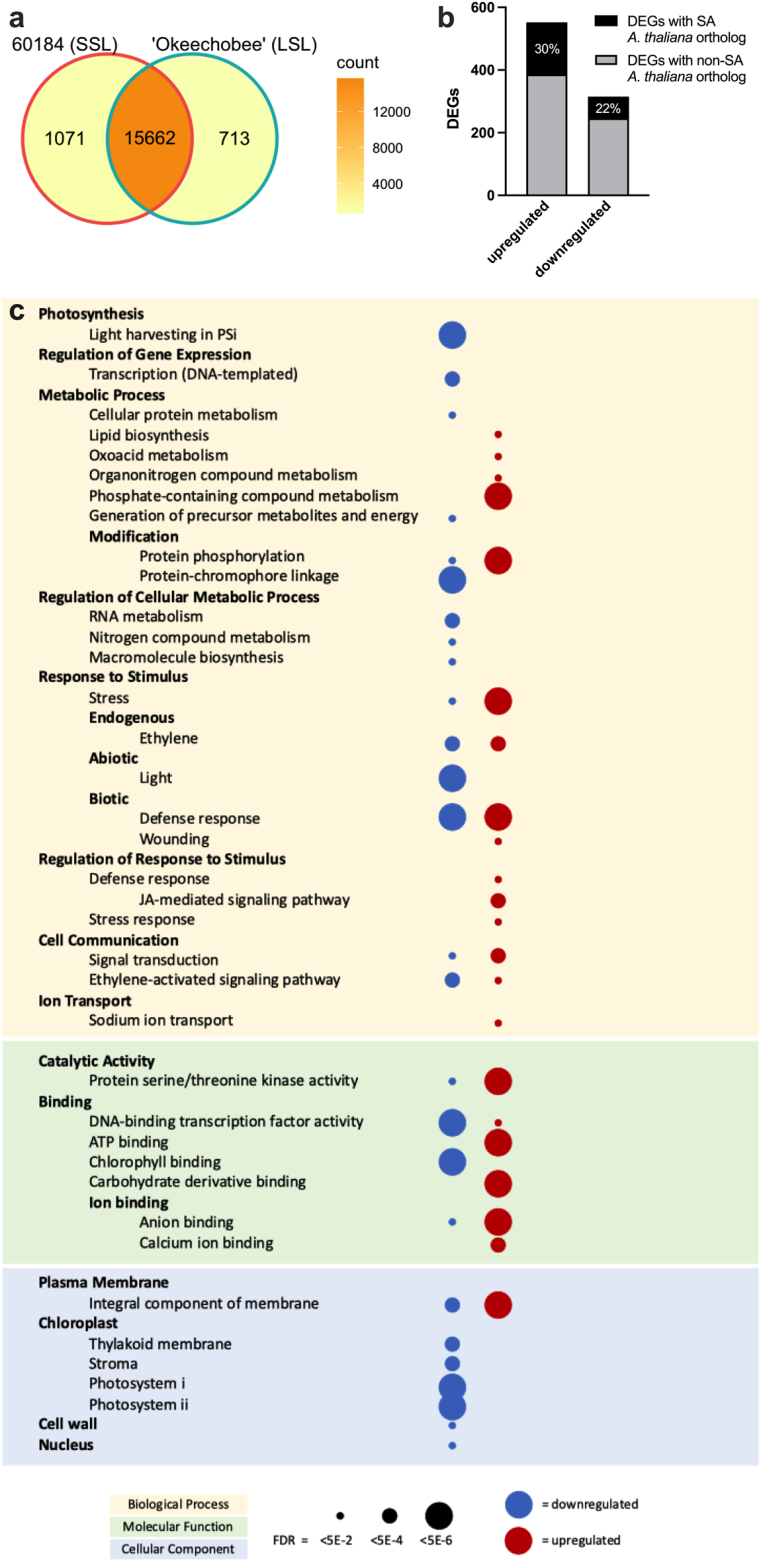
Distribution of expressed (TPM ≥ 100) genes among selected *L. sativa* genotypes, summary and GO enrichment analysis of DE genes between genotypes 60184 (SSL) and ‘Okeechobee’ (LSL). (**a**) Venn diagram of expressed genes between 60184 and ‘Okeechobee’. (**b**) Summary of DE genes in ‘Okeechobee’ relative to 60184. Black portion of bars denotes upregulated and downregulated *L. sativa* genes possessing an orthologous *A. thaliana* gene characterized as a SAG in the Leaf Senescence Database. (**c**) Significantly enriched GO terms in genes DE between 60184 and ‘Okeechobee’ at maturity. Enrichment in downregulated and upregulated genes is represented by blue dots and red dots, respectively. False discovery rate (FDR) shown by dot size. From the top, gene ontology categories are colored yellow (biological process), green (molecular function), and blue (cellular component). Related GO terms are grouped together based on the GO term hierarchy.

The majority (89.7%) of expressed genes (TPM ≥ 100) at maturity were expressed in both genotypes. There were 1071 and 713 genes uniquely expressed in genotypes 60184 (SSL) and ‘Okeechobee’ (LSL), respectively (Fig. 4a). In total, 868 characterized genes were DE (adjusted *p* < 0.05, |log_2_(FC)| ≥ 2) between 60184 (SSL) and ‘Okeechobee’ (LSL) genotypes at maturity. We found 552 upregulated genes and 315 downregulated genes (Fig. 4b).

Significantly enriched GO terms for specific biological processes, molecular functions, and cellular components were analyzed using the hierarchy feature of the Gene Ontology Resource. Enriched GO terms, KEGG pathways, and STRING local clusters for *A. thaliana* orthologs of lettuce DE genes were identified using the STRING database protein network tool (Fig. 4c). Among upregulated genes, monoterpene biosynthesis, JA biosynthesis, and protein autophosphorylation terms were enriched, and there was general enhancement of stress and defense-responsive genes. Terms for cell communication and signal transduction were also enriched. Anion binding and calcium binding, as well as sodium ion transport, were significantly overrepresented. Integral plasma membrane component was the only cellular component enriched term. STRING database analysis identified five overrepresented KEGG pathways supporting the GO enrichment analysis: plant–pathogen interaction, alpha-linoleic acid metabolism, plant hormone signal transduction, MAPK signaling pathway, and ABC transporters (Fig. 4c). Genes downregulated in ‘Okeechobee’ (LSL) were highly enriched for photosynthetic processes including light harvesting in photosystems I and II (PSI, PSII), protein-chromophore linkage, and chlorophyll binding, as well as for and light response and circadian rhythm. RNA metabolism and DNA-binding TF activity genes were overrepresented. Several terms describing response to ethylene and ethylene signaling pathways were enriched among downregulated genes. Additionally, regulation of abscisic acid biosynthesis and cellular response to gibberellins, two noted senescence-promoting phytohormones, were enriched (Fig. 4c).

### Identification of putative shelf-life associated genes

To identify novel shelf-life associated genes, we used our transcriptional data to find putative SAGs. Since no SAGs are formally characterized for lettuce in the Leaf Senescence Database^14^, we subset putative lettuce SAGs among DE genes between 60814 (SSL) and ‘Okeechobee’ (LSL). DE genes were screened for those possessing an *A. thaliana* orthologous gene documented as senescence-related in the Leaf Senescence Database. The full list of *A. thaliana* SAGs was merged with the list of lettuce DE genes and their *A. thaliana* orthologs. Among the DE genes, 30% of upregulated genes and 22% of downregulated genes had an *A. thaliana* senescence-associated ortholog (Fig. 4b). These subsets included 167 upregulated and 70 downregulated putative lettuce SAGs. The *A. thaliana* gene IDs of these subsets were analyzed using ShinyGO 0.76.1, and the top 20 enriched GO terms were ranked first by FDR < 0.5 and then fold enrichment (Supplementary Fig. S1). Among the upregulated lettuce putative SAGs, negative regulation of ethylene-activated signaling pathway and negative regulation of a phosphorelay signal transduction pathway were the most significantly enriched terms. Other enriched terms included several related to stress responses to external stimuli, including wounding, water deprivation, and acid chemical response. Several enriched terms were also related to endogenous hormone stimulus. The enrichment of terms related to negative regulation of ethylene and other signaling pathways suggests that ‘Okeechobee’ may have a suppressed response to ethylene, a senescence-promoting phytohormone, at this time in development. Perhaps the observed increased expression of stress and wounding responsive genes in ‘Okeechobee’ before harvest could indicate potential for a faster response to wounding at the time of harvest that allows for an extended shelf-life.

Among downregulated genes, the top enriched GO terms included response to red light, circadian rhythm, and phosphorelay signal transduction system. Terms related to external biotic stimulus response were also enriched, with response to bacterium being the most specific enriched term in this hierarchy. There were also several enriched terms related to endogenous hormone stimulus response (Supplementary Fig. S1). It is interesting to note that red light response-related genes are enriched among those expressed at lower levels at the preharvest time point in ‘Okeechobee’, as a decline in expression of chloroplast and photosynthesis-related genes is a hallmark of promotion of postharvest senescence. Additionally, considering that genes with phosphorelay transduction pathway and hormone response functions were enriched among both upregulated and downregulated DE genes, these results suggest that various phytohormones are differentially regulated between 60184 and ‘Okeechobee’.

**Figure 5:**
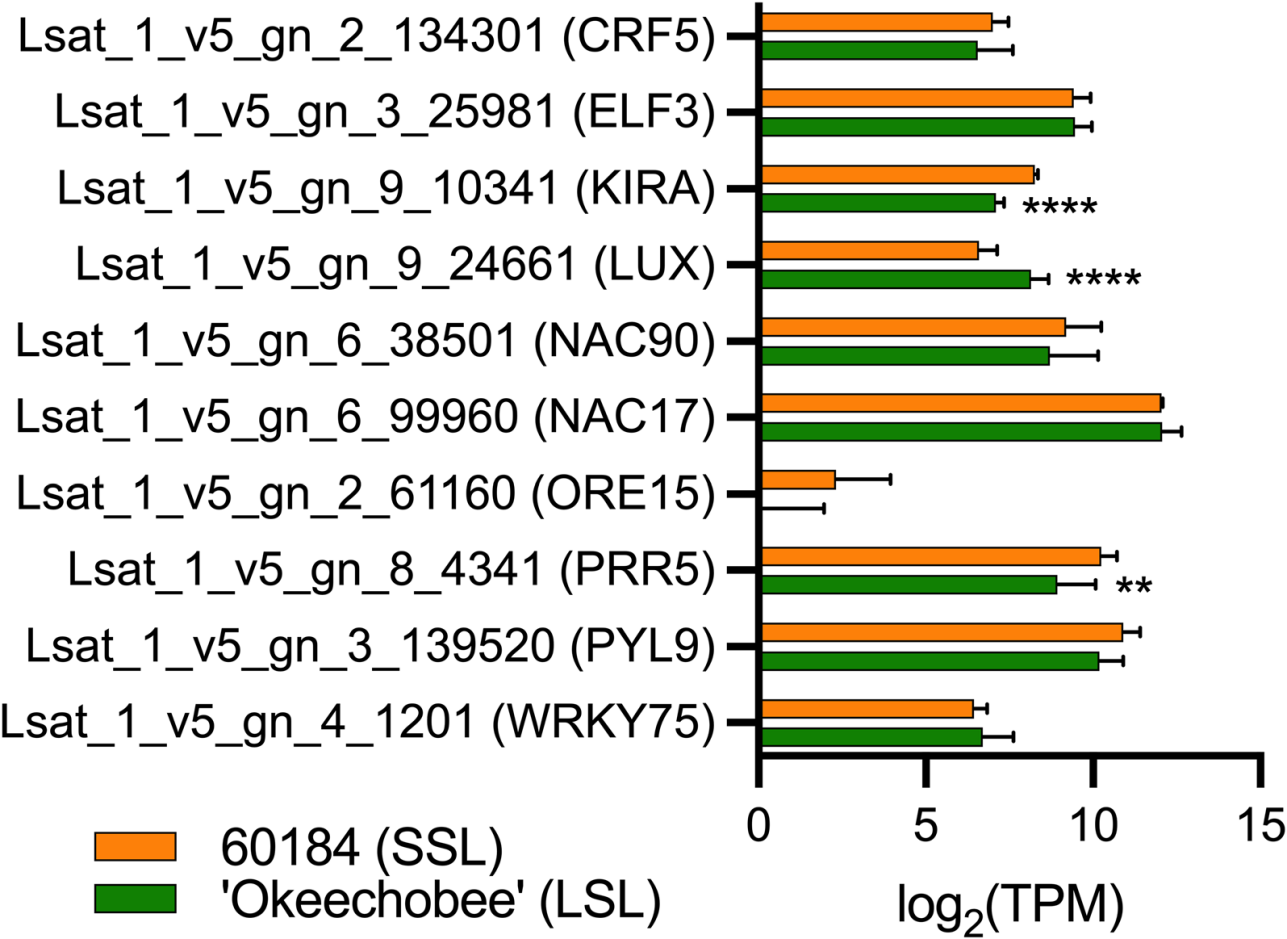
Identification of pre-harvest markers as putative lettuce SAGs. Expression of ten *L. sativa* putative SAGs based on the Wang et al.^15^ set in 60184 (SSL) and ‘Okeechobee’ (LSL) genotypes at mature preharvest developmental stage, presented as log_2_(TPM) values. Asterisks indicate significant difference in expression relative to 60184. *p < 0.05, **p < 0.01, ***p < 0.001, ****p < 0.0001.

Additionally, we specifically examined the expression patterns of lettuce orthologs of ten previously characterized *A. thaliana* SAGs^15^. RNA-seq expression data for this subset of putative lettuce SAGs was extracted to compare gene expression levels during the preharvest developmental stage with characterized postharvest trends. Several genes among this subset were DE between genotypes at maturity. *LsPRR5* and *LsKIRA* had significant downregulation and *LsLUX* was significantly upregulated in ‘Okeechobee’ (LSL) relative to 60184 (SSL) (Fig. 5). *AtPRR5* (*LsPRR5* ortholog) and *AtKIRA* are known to be positive regulators of senescence^16,17^, while *AtLUX* is a negative regulator of senescence^18^. The other seven genes were not significantly DE at maturity. These results suggest that *LsPRR5*, *LsKIRA*, and *LsLUX* expression could be potential indicators of postharvest shelf-life, although it is important to consider that there are likely dynamic changes in SAG expression occurring with the onset of postharvest senescence, so markers used to predict shelf-life in lettuce may vary with developmental stage.

### Senescence-associated TFs represent a large portion of DE genes between genotypes

**Figure 6:**
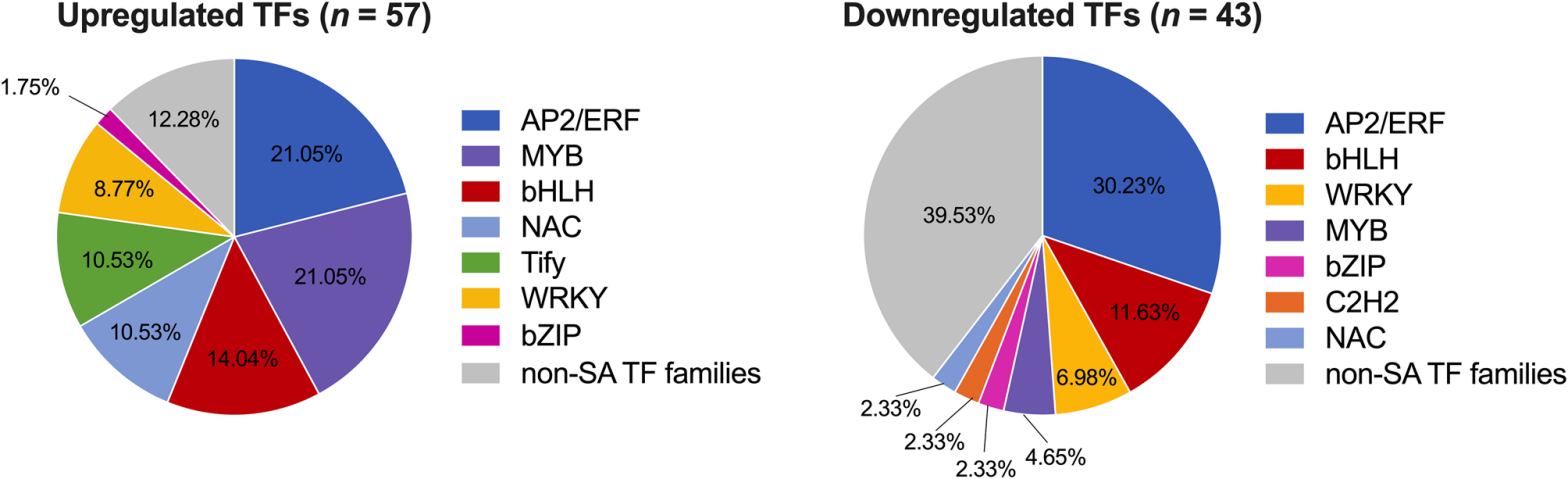
TF families among DE genes in ‘Okeechobee’ (LSL) relative to 60184 (SSL). Colored wedges represent TF families largely noted in literature as comprising SAGs. *AP2/ERF* APETALA2/ethylene responsive factors, *bHLH* basic helix-loop-helix, *NAC* (NAM, ATAC, CUC), *bZIP* basic leucine zipper, *C2H2* cys2-his2 zinc finger.

A large portion of characterized SAGs are TFs. TFs among DE genes were mined by sub-setting lettuce DE genes with an *A. thaliana* ortholog listed in the Plant TF Database^19^. Functional “deflines” from the Phytozome V13 *L. sativa* genome resource were also checked to validate the TFs identified using ortholog descriptions. We found 57 TFs with upregulated expression and 43 TFs with downregulated expression in ‘Okeechobee’ (LSL) (Fig. 6). Among all DE TFs, 76% belonged to families previously characterized as senescence-associated in the literature^20^, including the APETALA2/ETHYLENE RESPONSIVE FACTOR (AP2/ERF), NAC (NAM, ATAC, CUC), MYB, and WRKY families, and other less common TF families (Fig. 6). AP2/ERF TFs represented the largest proportion of all DE TFs, accounting for 21.05% of upregulated TFs and 30.23% of downregulated TFs in ‘Okeechobee’ (LSL). Members of this family including several ERFs and dehydration-responsive element-binding protein (*DBP*) genes were found in both upregulated and downregulated gene sets. Several members of the WRKY and NAC TF families previously characterized as negative regulators of senescence were found among genes upregulated in ‘Okeechobee’ (LSL), including *LsNAC83, LsWRKY48, LsWRKY70,* and *LsWRKY57*^6,21,22^. We identified the TIFY family, an interesting gene family in which its members were exclusively upregulated in ‘Okeechobee’ (LSL). The TIFY family is a plant-specific TF family characterized by the conserved motif TIF[F/Y]XG and comprises four sub-families, including the *JAZ*^23^. Six *JAZ* genes were upregulated in ‘Okeechobee’. These proteins repress jasmonate signaling, and JA is a positive regulator of leaf senescence^6^. These results suggest several putative negative regulators of lettuce leaf senescence belong to NAC, WRKY, and TIFY TF families.

### Genes regulating JA biosynthesis and signaling are upregulated in LSL genotype

**Figure 7:**
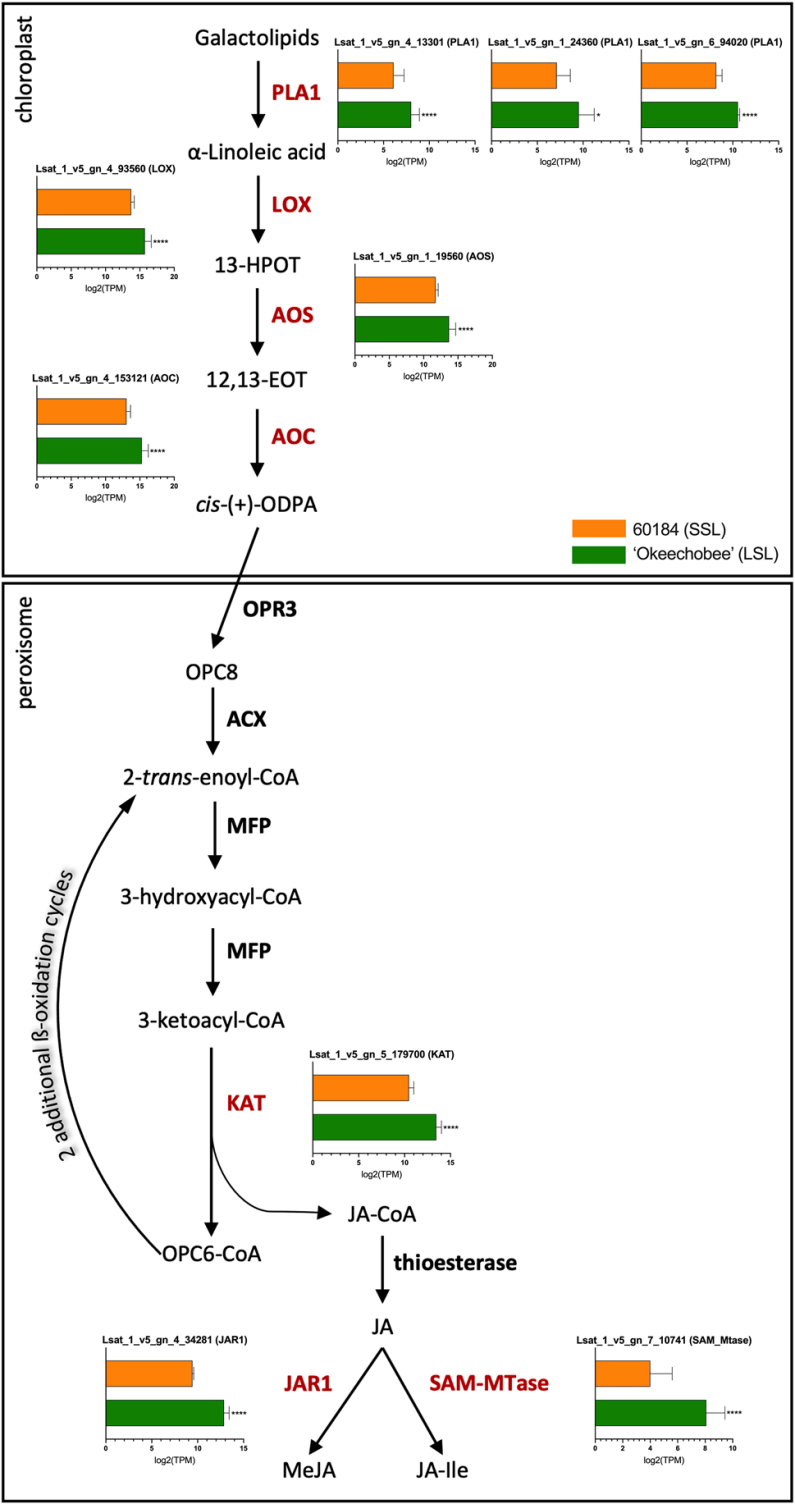
Differential expression of lettuce JA biosynthesis pathway genes in lettuce genotypes at maturity. Synthesis of JA-Ile and MeJA from galactolipid precursors. Red color indicates significant upregulation in ‘Okeechobee’ (LSL). Graphs accompanying DE genes show expression of JA biosynthesis genes in 60184 (SSL) and ‘Okeechobee’ genotypes at mature preharvest developmental stage, presented as log_2_(TPM) values. Asterisks indicate significant difference in expression relative to 60184. *p < 0.05, **p < 0.01, ***p < 0.001, ****p < 0.0001. *PLA1* phospholipase A_1_, *LOX* lipoxygenase, *13-HPOT* (13S)-hydroperoxyoctadecatrienoic acid, *AOS* allene oxide synthase, *12,13-EOT* 12,13-epoxyoctadecatrienoic acid, *AOC* allene oxide cyclase, *ODPA* 12-oxophytodienoic acid, *OPR3* OPDA reductase 3, *OPC8* 3-oxo-2(2′[Z]-pentenyl)-cyclopentane-1-octanoic acid, *ACX* acyl-CoA oxidase, *MFP* multifunctional protein, *KAT* 3-ketoacyl-CoA thiolase, *JAR1* JA-amino acid synthetase, *SAM-MTase* *S*-adenosyl-l-methionine-dependent methyltransferase, *JA* (+)-7-iso-jasmonic acid, *JA-Ile* jasmonoyl-isoleucine, *MeJA* methyl jasmonate.

Significant enrichment of genes involved in JA biosynthesis and regulation of JA hormone signaling was discovered among genes upregulated in ‘Okeechobee’ (LSL) compared with 60184 (SSL) at preharvest. Many genes functioning in the pathways for jasmonoyl-isoleucine (JA-Ile) and methyl jasmonate (MeJA) synthesis^24^ were significantly upregulated, including genes encoding phospholipase A1 (*LsPLA1*), lipoxygenase (*LsLOX*), allene oxide synthase (*LsAOS*), allene oxide cyclase (*LsAOC*), 3-ketoacyl-CoA thiolase (*LsKAT*), jasmonic acid-amino acid synthetase (*LsJAR1*), and *S*-adenosyl-l-methionine-dependent methyltransferase (*LsSAM-MTase*) (Fig. 7b).

**Figure 8:**
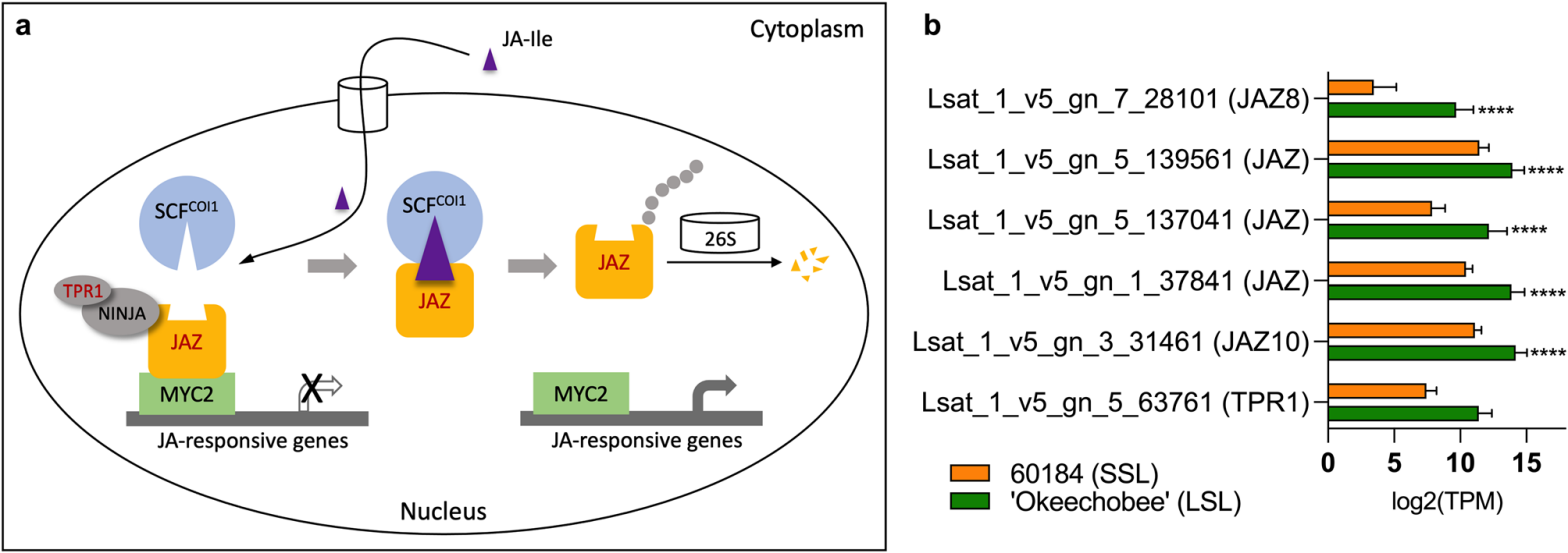
Differential expression of lettuce JA signaling pathway genes in lettuce genotypes at maturity. (**a**) Signaling pathway of JA-Ile. Red color indicates significant upregulation in ‘Okeechobee’ (LSL). *JA-Ile* jasmonoyl-isoleucine, *SCF* Skp1 cullin and F-box proteins, *COI1* coronatine insensitive 1, *JAZ* jasmonate ZIM-domain protein, *NINJA* NOVEL INTERACTOR OF JAZ, *TPR1* TOPLESS-RELATED1 protein, *26S* 26S proteasome. (**b**) Differential expression of JA-Ile signaling pathway genes in 60184 (SSL) and ‘Okeechobee’ genotypes at maturity, presented as log_2_(TPM) values. Asterisks indicate significant difference in expression relative to 60184. *p < 0.05, **p < 0.01, ***p < 0.001, ****p < 0.0001.

In addition to upregulation of JA biosynthesis genes, several genes regulating JA signal transduction were also upregulated in ‘Okeechobee’ (LSL). This included five genes of the *JAZ* family (Fig. 8b). The lettuce ortholog of *A. thaliana* TOPLESS-RELATED1 (*TPR1*), whose protein product acts as a JAZ co-repressor with NOVEL INTERACTOR OF JAZ (NINJA) protein^25^, was significantly upregulated in ‘Okeechobee’ (LSF). While there is increased expression of JA-signaling repressor genes in ‘Okeechobee’ (LSF), there is also significant upregulation of *LsMYC2*, albeit below our log_2_(FC) threshold. Taken together, these results suggest that levels of JA biosynthesis and JA-signal transduction may have an influence on variation in the onset of senescence and thusly affect shelf-life.

### Cell wall modification-associated genes are DE between genotypes

**Figure 9:**
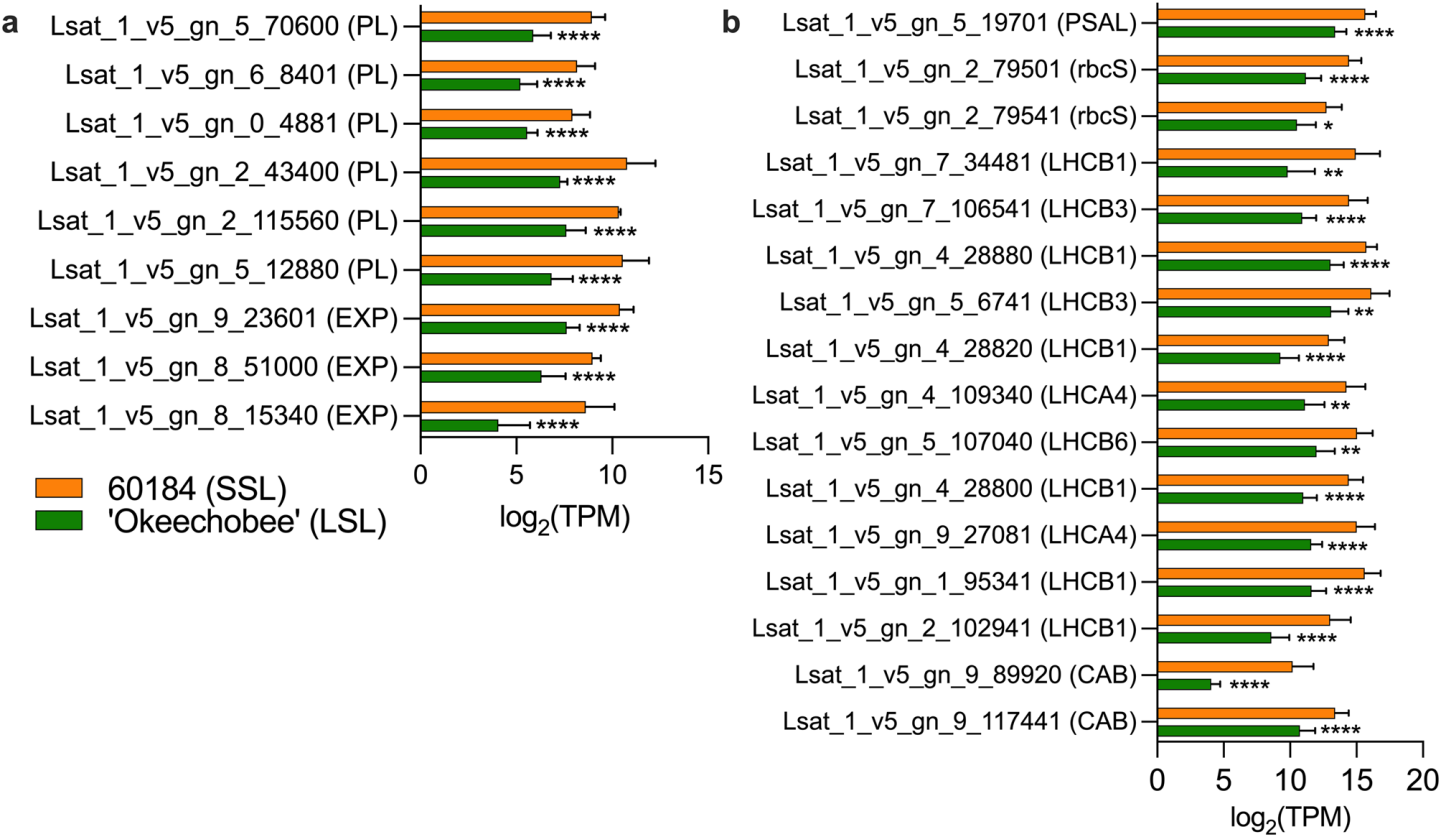
Relative expression levels of chloroplast-associated and cell wall-associated DE genes in lettuce genotypes at maturity. (**a**) Relative expression of cell wall-associated and (**b**) chlorophyll and photosynthesis-associated downregulated genes in 60184 (SSL) and ‘Okeechobee’ (LSL) genotypes, presented as log_2_(TPM) values. Asterisks indicate significant difference in expression relative to 60184. *p < 0.05, **p < 0.01, ***p < 0.001, ****p < 0.0001.

Modifying the traits of lettuce cell walls to reduce loosening has been shown to extend shelf-life by decreasing membrane leakage and increasing leaf strength^8^. Therefore, we investigated the relative expression of cell wall modification and degradation genes. Transcripts mapped to 27 *LsXTH* genes, 25 of which were not DE between the two genotypes and two of which were upregulated in ‘Okeechobee’ (LSL). In ‘Okeechobee’ (LSL), six lettuce *PL* genes and three lettuce *EXP* genes were downregulated (Fig. 9a). The higher level of expression of these genes in 60184 (SSL) suggests that this genotype may have looser cell walls or an earlier onset of cell wall degradation than ‘Okeechobee’ (LSL), which could be contributing factors to a more rapid postharvest senescence and shortened shelf-life.

### Chlorophyll- and photosynthesis-associated genes are downregulated in LSL genotype

Among organelles, chloroplasts are highly modulated by senescence and stress response processes. The chloroplast is the site of early enzymatic degradation during senescence, and breakdown of chlorophyll is one of the major sources of quality loss in lettuce during shelf-life storage^26^. Chlorophyll binding protein genes and other chloroplast-associated genes were highly significantly enriched among downregulated DE genes in ‘Okeechobee’ (LSL) relative to 60184 (SSL). We found that thirteen light harvesting (*LsLHCB*) and *LsCAB* protein genes, two ribulose bisphosphate carboxylase small subunit (*LsrbcS*) genes, and a photosystem I subunit V (*LsPSAL*) gene were downregulated (Fig. 9b). Transcripts were mapped to two chlorophyllase genes, one of which was minimally expressed in both genotypes, while the other was significantly upregulated in ‘Okeechobee’ (LSL). No *LsLHCB* or *LsCAB* genes were found to be upregulated in ‘Okeechobee’ (LSL). This result suggests a reduction in photosynthetic activity in ‘Okeechobee’ (LSL) relative to 60184 (SSL) at preharvest.

### Postharvest evaluation of transcriptional expression of selected DE genes

**Figure 10:**
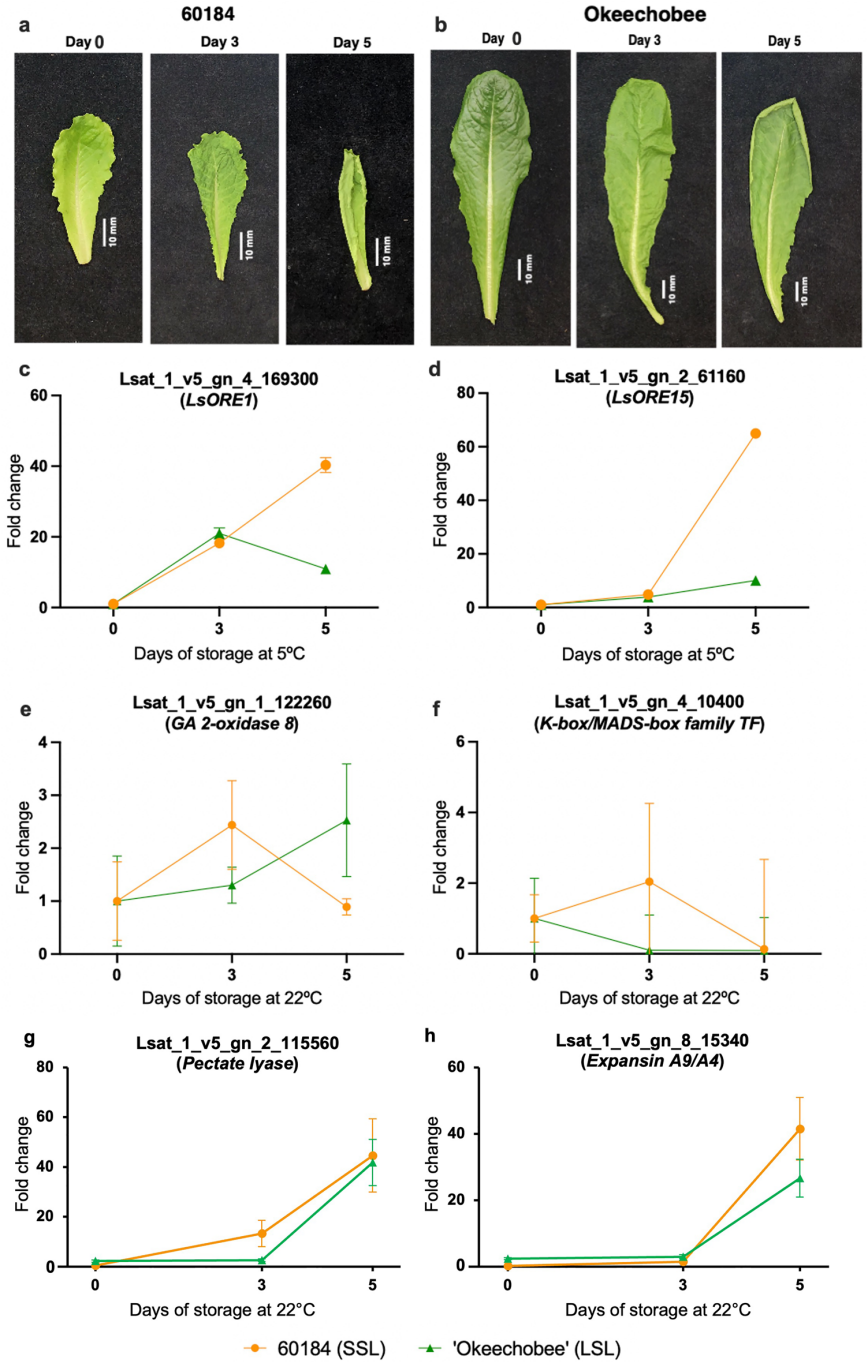
Validation of preharvest markers to identify postharvest traits. (**a**,**b**) Leaf shrinkage in detached leaves of (**a**) experimental line 60184 (SSL) and (**b**) ‘Okeechobee’ (LSL) genotypes at 0, 3, and 5 days of storage at room temperature. Scale bar = 10 mm. (**c**,**d**) Relative expression of senescence molecular markers (**c**) *LsORE1* and (**d**) *LsORE15* in detached leaves of experimental line 60184 (SSL) and ‘Okeechobee’ (LSL) genotypes during 5 days of storage at 5 °C*.* (**e**–**h**) Relative expression of RNA-seq candidate genes (**e**) Lsat_1_v5_gn_1_122260 (*GA 2-oxidase 8*), (**f**) Lsat_1_v5_gn_4_10400 (*K-box/MADS box family TF*), (**g**) pectate lyase (Lsat_1_v5_gn_2_115560) and (**h**) Expansin A9/A4 (Lsat_1_v5_gn_8_15340) in detached leaves of experimental line 60184 (SSL) and ‘Okeechobee’ (LSL) genotypes during 5 days of storage at 22 °C.

To further validate our approach to identifying molecular markers for preharvest factors linked to postharvest traits for prolonged shelf, we harvested leaves from ‘60184’ a SSL and ‘Okeechobee’ a LSL lettuce genotype (Fig. 10). Leaf shrinkage and dehydration were observed after stored at room temperature for 3 days and 5 days (Fig. 10a,b). First, we used *LsORE1* and *LsORE15* as molecular markers since they have been reported as senescence associated genes (SAGs) in Arabidopsis^17,27^. We performed time-course RT-qPCR to test the expression of both SAGs on lettuce harvested after 3 days and 5 days using leaf samples from the experimental line 60184 (SSL) and ‘Okeechobee’ (LSL) stored at 5 °C and 95–98% relative humidity to simulate commercial conditions. We found that the expression of both, *LsORE1* (Fig. 10c) and *LsORE15* (Fig. 10d), was significantly induced after postharvest in 60184 (SSL), corroborating that the short shelf-life of 60184 (SSL) is due to the acceleration of senescence. We also selected two highly up-regulated genes in the SSL genotype, GA 2-oxidase 8 (Lsat_1_v5_gn_1_122260) and the transcription factor K-box/MADS-box family (Lsat_1_v5_gn_4_10400) and performed a similar transcriptional analysis at 1, 3, and 5 days postharvest stored at 22 °C and 60–70% relative humidity to simulate accelerated senescence (Fig. 10c,d). Consistent with our results, both GA 2-oxidase 8 and K-box/MADS-box family genes were induced in ‘60184’ (SSL) at day 3 after harvest compared with ‘Okeechobee’ (LSL). Additionally, we selected a pectate lyase (Lsat_1_v5_gn_2_115560) which transcriptional expression was dramatically induced at day 3 after harvest in 60184 (SSL) with a greater than 16-fold increase (Fig. 10g). In contrast to strong increase in 60184 (SSL) at day 3 after harvest, the change of expression in Lsat_1_v5_gn_2_115560 gene was not detected in ‘Okeechobee’ (LSL) cultivars (Fig. 10g). Although the expression of Lsat_1_v5_gn_2_115560 significantly induced in both 60184 (SSL) and ‘Okeechobee’ (LSL) cultivars at day 5, the greater expression was found in 60184 (SSL). These results suggest that a pectate lyase gene in 60184 backgrounds may rapidly respond to postharvest stresses and trigger the early senescence. Consistent with these results, we examined the expression of another gene encoded an expansin A9/A4 (Lsat_1_v5_gn_8_15340), and found it highly induced in 60184 (SSL) at day 5 of postharvest conduction with greater than 2-fold increase to ‘Okeechobee’ (LSL) cultivars (Fig. 10h). These data support the possible role of pectate lyase and expansin as a regulator in response to postharvest stresses and potentially lead to variability of shelf-life in 60184 (SSL) and ‘Okeechobee’ (LSL) cultivars.

## Discussion

Using leaf morphohistological analyses coupled with a transcriptomic approach, we identified potential genetic markers linked to shelf-life. Our analyses suggest that several leaf developmental characteristics previously found to correlate with extended lettuce shelf-life^13^ may not apply to all lettuce cultivar types. Therefore, other strategies including the identification of genetic factors to establish preharvest markers of postharvest shelf-life phenotype are needed.

We found that ‘Okeechobee’ (LSL) had significantly lower stomatal index than the breeding line 60184 (SSL). While this suggests that stomatal index may correlate with shelf-life in these romaine genotypes, we found little difference in other leaf histological characteristics, including ECA and stomatal density, which have been previously correlated with shelf-life^13^. 60184 (SSL) had significantly larger lamina thickness than ‘Manatee’ (ISL) and ‘Okeechobee’ (LSL) (Fig. 2b). It was hypothesized that lamina thickness would be relatively larger in a lettuce genotype with longer shelf-life, but this was not the case. Higher leaf lifespan is found across many plant species that had high leaf mass per unit area due to thick leaf lamina or high cellular density in leaf tissue^9^. These relationships between leaf developmental traits and shelf-life were demonstrated in ‘Lollo Rosso’^13^ and in a recombinant inbred line between a crisphead lettuce and wild lettuce (*L. serriola*)^7^. There is a high amount of morphological and genetic variation that differentiates the horticultural types of *L. sativa*, such that each type forms a distinct cluster when the population of cultivated lettuce is examined^28^. ‘Lollo Rosso’, a loose-leaf lettuce, and crisphead lettuces have vastly different morphologies and showed similar relationships between leaf developmental characteristics, so it is interesting that this ideotype does not appear to hold true for most characteristics of these selected romaine lettuces.

The lettuce ideotype for extended shelf-life includes small cell area that maximizes strong cell wall surface area^13^. We examined the expression of cell wall-associated genes between 60184 (SSL) and ‘Okeechobee’ (LSL) romaine lettuces and found downregulation of several genes related to cell wall integrity in ‘Okeechobee’ (Fig. 9a). Investigations of the relationships between leaf cell wall characteristics and shelf-life in lettuce have largely focused on the cell wall loosening role of *LsXTH* genes^29^. Downregulation of *LsXTH* genes in ‘Okeechobee’ relative to 60184 (SSL) was hypothesized, consistent with its longer shelf-life, but we observed no difference in expression of the majority of *LsXTH* mapped genes, with only two upregulated *LsXTH*s in ‘Okeechobee’ (LSL). In contrast, we found downregulation of six *PL* and three *EXP* genes in the LSL cultivar (Fig. 9a). EXPs contribute to cell enlargement by loosening polysaccharide networks in the cell wall, and PLs are known to contribute to cell wall degradation during fruit ripening and senescence^30^. *PL* knockout mutants in the tomato cultivar ‘Ailsa Craig’ and *PL* antisense knockdown mutants in strawberry both resulted in decreased PL activity and produced firmer fruits^31,32^. Additionally, upregulation of *PL* genes and *EXP1* caused by *SlFSR* overexpression has been correlated with shortened shelf-life in tomato^33^. Recent cell wall structure discoveries have revised the conventional model, wherein pectin-Ca^2+^ gel matrices exist independently of the cellulose-hemicellulose network, proposing that pectin plays a more integral role by directly cross-linking with cellulose microfibrils^34^. This newer model is used to interpret the large impact of pectin degradation enzymes on fruit softening^32^. Considering this proposed structural influence of PL and our observed results, *PL* expression may be a candidate for a preharvest marker of lettuce shelf-life.

Our analysis also identified lettuce genes that have orthologous previously characterized in *A. thaliana* as SAGs (Fig. 4b, Supplementary Fig. S1), supporting the idea that the genes in the current study and other DE genes, may be putatively senescence-associated, and may serve as preharvest markers of postharvest senescence in lettuce. Notably, the majority of which were members of senescence-associated TF families (Fig. 6). There were several lettuce genes among the upregulated TFs whose role in senescence has been previously investigated in other species. For instance, *LsNAC83, LsWRKY48,* and *LsWRKY70* had increased expression in ‘Okeechobee’ (LSL) and their orthologous genes in strawberry were upregulated during the storage of long-shelf-life^22^. Also, Arabidopsis orthologous *AtWRKY70* and *AtNAC83* delayed senescence^6^, and *AtWRKY57* act as negative regulator of JA-induced leaf senescence^21^. These findings are consistent with our results showing upregulation of these genes in ‘Okeechobee’ (LSL).

Our RNA-seq analysis found many genes related to JA biosynthesis and JA signaling to be significantly upregulated in the LSL cultivar ‘Okeechobee’. Increased expression of JA biosynthesis genes *LsPLA1, LsLOX, LsAOS, LsAOC, LsKAT, LsJAR1, and LsSAM-MTase* in ‘Okeechobee’ suggest a higher endogenous level of JA in this cultivar at the preharvest stage (Fig. 7b). Endogenous levels of JA are known to be four-fold greater in senescent *A. thaliana* leaves than non-senescent ones, and exogenous applications of MeJA have been shown to promote senescence in detached *A. thaliana* leaves^35^. This is opposite of the hypothesis that the LSL cultivar may produce less JA in the early stages of senescence. In addition to increased JA biosynthesis in ‘Okeechobee’, our analysis also showed evidence of differences in JA signaling between the SSL and LSL genotypes. Under pre-harvest conditions, endogenous JA levels are low^36^, and *JAZ* genes function as key repressors in the JA signal transduction pathway by interacting with MYC2, a master regulator of downstream JA signaling cascades. During stress or wounding response (postharvest), JA levels accumulate, and *JAZ* repressors are degraded in the 26S proteasome via *COI1*-mediated ubiquitination^24,37^. The master regulator *MYC2* is then expressed and promotes downstream expression of JA response program genes (Fig. 8a)^38^. The significant upregulation of several *LsJAZ* repressors and *LsTPR1*, a JAZ corepressor, in ‘Okeechobee’, suggests stronger repression of the JA signaling pathway relative to 60184 (Fig. 8b). In previous studies, *A. thaliana* overexpressing *JAZ4* and *JAZ8* had a delayed senescence relative to the wild type when treated with MeJA^21^. *JAZ4* and *JAZ8* was shown to interact directly with the TF *WRKY57* to negatively regulate senescence^21^. *LsJAZ8* and *LsWRKY57* were identified among upregulated genes in ‘Okeechobee’ in agreement with the delayed senescence phenotype. The observed upregulation of these and other *LsJAZ* genes could reflect an important role of JA signaling repression in delaying the onset of postharvest senescence in lettuce, but this observation must also be reconciled with the observed increased biosynthesis of JA, which would otherwise promote senescence. Previous research has found a similar pattern of upregulated JA biosynthesis and JA signaling genes at 23–25 days after seeding, including both *JAZ* genes and *MYC2*^39^*.* This time point may represent a preharvest developmental stage similar to the maturity time point at which lettuce gene expression was evaluated in this study. Phased increases in JA from 21–39 days after seeding have been found previously^39^, suggesting there are dynamic changes in JA levels leading up to and during senescence.

Postharvest is a critical stage that requires a well antioxidant balance to maintain quality after senescence. A moderate antioxidant level is often beneficial to signal various crucial steps during growth and development, including ripening and increased resistance in a postharvest condition. However, elevated antioxidant levels that are not coupled with high antioxidant capacity often poses great risk to the plant tissues^40^. Notably, the biosynthesis of both JA-Ile and MeJA was upregulated in ‘Okeechobee’, as implicated by the upregulation of *LsJAR1* and *LsSAM-MTase,* respectively*.* MeJA is an elicitor that induces oxidative stress, enzymatic antioxidant activity, and secondary metabolite accumulation in fruit and vegetable^41^. *LsJAR1* and *LsSAM-MTase* are responsible for catalyzing the final step to form the jasmonate derivatives JA-Ile and MeJA, respectively (Fig. 7a)^42,43^. Upregulation of these genes suggests that higher endogenous levels of jasmonate production might be accumulated at preharvest in ‘Okeechobee’ (LSL) leaf tissue. Exogenous applications of MeJA have been shown to increase the antioxidant capacity and delay the senescence of many fruits and vegetables^44^. Significant increases in total phenolic content and antioxidant capacity after preharvest MeJA treatments of romaine lettuces^45^, and similar results were reported after postharvest MeJA treatment of wounded crisphead lettuce^46^. Paired with upregulation of MeJA biosynthesis genes in ‘Okeechobee’, we also found enrichment of GO terms relating to terpene biosynthesis, defense response, and stress response among upregulated genes (Fig. 4c). This suggests a stronger antioxidant capacity and response in ‘Okeechobee’ (LSL) than 60184 (SSL), which could contribute to its delayed senescence and extended shelf-life. Early stages of senescence in detached lettuce leaves have been characterized as a stress response with early upregulation of antioxidant activity^3^, proposing that this activity may delay senescence onset. This program appears consistent with the gene expression seen at the preharvest time point in ‘Okeechobee’ (LSL), suggesting that this cultivar’s inherent antioxidant capacity may contribute to its postharvest shelf-life. Further studies to quantify endogenous jasmonate levels among lettuce genotypes and between senescent and non-senescent tissue are needed to fully elucidate the role of JA levels in lettuce postharvest senescence.

In this study, we identified several functional groups and specific genes whose DE patterns between LSL and SSL genotypes may serve as preharvest markers of lettuce postharvest shelf-life and be used for selection in lettuce breeding programs. JA signaling repression, antioxidant capacity, and PL activity during preharvest are proposed as predictors of lettuce postharvest shelf-life. TFs *LsNAC83, LsWRKY48, LsWRKY70*, and *LsWRKY57* are putatively senescence-associated in lettuce and could also act as indicators of extended shelf-life. Our results propose that these genes could potentially be used as preharvest markers of postharvest senescence onset and predict shelf-life in lettuce. These markers could be validated in a larger set of lettuce germplasm with known shelf-life.

## Materials and methods

### Plant material

Three romaine lettuces (*Lactuca sativa* var. Longifolia) including an experimental breeding line, 60184 and the commercial cultivars “Manatee”, and “Okeechobee” (LSF) were grown in Belle Glade, FL and used for leaf tissue histological analysis (Fig. 1a–c). These genotypes were selected from a larger set of romaine genotypes based on genotypic variability in their shelf-life^47^. Breeding line 60184 has a relatively short shelf-life (SSL), ‘Manatee’ has an intermediate shelf-life length (ISL), and ‘Okeechobee’ has a longer shelf-life (LSL) (Fig. 1d,e).

### Shelf-life determination

To determine shelf-life length, experiments, lettuce heads were harvested approximately 60 days after planting. Lettuce heads were collected and stored at 15 °C, as previously described^47^. Lettuce shelf-life was evaluated using a rating scale of 0 to 9, where 9 = lettuce heads free of damage and 0 = lettuce heads completely decayed. Evaluations were made according to the storage temperature; lettuce stored at 15 °C was evaluated at 1, 3, 5 and 7 days after initial storage.

### Leaf tissue morphological analysis

Epidermal cell area (ECA) and lamina thickness were analyzed in tissues extracted from the same lettuce plants as those used for RNA extraction and sequencing. ECA was analyzed using a modified version of the protocol described by Zhang et al.^7^. At maturity, a 10 mm leaf disc was excised from the first fully expanded leaf counting outwards from the rosette center using a hole punch, located ~ 20 mm from the leaf apex and parallel to the midrib vein (*n* = 4 per genotype). Clear nail varnish was applied to the adaxial surface of the leaf and allowed to dry for ~ 15 min. The varnished side of the disc was pressed onto sticky tape, and the disc was peeled away to leave a residual imprint. The tape was applied to a glass microscope slide, and imaging was conducted using a Leitz Laborlux S light microscope (Leica Camera, Wetzlar, Germany) at 100 × magnification. Using ImageJ software^48^ for analysis, image area was divided by the total number of cells within the image area to obtain the mean ECA. Total stomates in the image were counted to calculate stomatal density as stomata per square millimeter, and stomatal index was calculated as stomatal index (%) = [stomata]/[total cells + stomata] × 100.

Lamina thickness for each lettuce genotype was measured from transverse leaf sections. At maturity, a 1.5 cm^2^ rectangle of tissue with the leaf midvein centrally positioned was excised from the first fully expanded leaf counting outwards from the rosette center. Tissue fixation, clearing, and embedding were conducted based on the protocol developed by Begcy and Walia^49^. Tissues were fixed in a Formaldehyde Alcohol Acetic Acid (FAA) fixative (10% formaldehyde, 5% glacial acetic acid, 50% ethanol, 35% deionized water) and dehydrated in an ethanol series. Tissues were cleared in a series of ethanol and xylene mixtures and then embedded in paraffin wax. Transverse sections of 10 µm thickness (*n* = 12 per genotype) were mounted on glass slides and stained with 0.004% toluidine blue^50^. Imaging was conducted using a Leitz Laborlux S light microscope (Leica Camera, Wetzlar, Germany) at 10 × magnification. Images of the transverse sections of the lamina were taken lateral to the midvein, and ImageJ software^48^ was used to quantify average lamina thickness from measurements at three points spaced across the image.

### Tissue collection, RNA extraction, and RNA sequencing

To analyze the global transcriptional gene expression, we selected two contrasting lettuce genotypes with variable shelf-life. We collected the first fully expanded leaf counting outwards from the rosette center from mature romaine genotypes 60184 (SSL) and ‘Okeechobee’ (LSL). For RNA extraction, lettuce plants were grown in 4-inch pots containing PRO-MIX HP media (PRO-MIX, Quakertown, PA, USA) in a growth chamber maintained with a 16 h day light cycle at 22 °C. Upon maturity, RNA was extracted using the Nucleospin RNA Plant and Fungi kit (Machery-Nagel, Düren, Germany) with on-column DNase treatment, and initial quantification and quality was checked on a NanoDrop OneC spectrophotometer (Thermo Fisher Scientific, Waltham, MA, USA). Next generation sequencing of the extracted RNA samples was conducted at the University of Florida Interdisciplinary Center for Biotechnology Research (ICBR) Gene Expression and Genotyping Core Facility (Gainesville, FL, USA). RNA quality was checked using a 2100 Bioanalyzer (Agilent Technologies, Santa Clara, CA, USA) and poly-A RNA-seq libraries were prepared following Illumina protocols (Illumina, San Diego, CA, USA). Paired end RNA sequencing (RNA-seq) was performed using an Illumina NovaSeq6000 with a read length of 2 × 150 base pairs (bp).

### RNA-seq and pathway enrichment analyses

Reads acquired from the Illumina NovaSeq 6000 platform were trimmed and filtered with the Cutadapt program^51^ by removing sequencing adaptors and low-quality bases with a quality phred-like score less than 20. Reads shorter than 60 bp were excluded from RNA-seq analysis. The genome of *Lactuca sativa* (version: Lsativa_467_v5) from Joint Genome Institute (Berkeley, CA, USA) was used as the reference genome for RNA-seq analysis. The cleaned reads of each sample were individually mapped to the reference sequences using the Spliced Transcripts Alignment to a Reference (STAR) package (v2.7.9a)^52^. The mapping results were processed with the High-Throughput Sequence Analysis (HTSeq) in Python (v0.11.2)^53^, SAMtools, and by using scripts developed in-house at ICBR to remove potential polymerase chain reaction (PCR) duplicates and select and count uniquely mapped reads for gene expression analysis. Principal component analysis (PCA) analysis was performed in R to detect outlier samples among all the identified genes. The counted reads of each gene were then analyzed by a DESeq2-based R pipeline^54^. Differentially expressed (DE) genes were analyzed in a pairwise comparison between the breeding line 60814 (SSL) and ‘Okeechobee’ (LSL) samples. Transcripts per million (TPM) counts were averaged across biological replicates and genes were filtered for moderate expression (TPM ≥ 100) in at least one of the two genotypes. Moderately expressed genes were then filtered for an adjusted *p* < 0.05 and |log_2_(FC)| ≥ 2. UniProt IDs and *A. thaliana* ortholog gene identifiers for DE genes, as well as Phytozome V13 lettuce gene “auto defline” annotations, were extracted using BioMart through Phytozome V13^55,56^. Uncharacterized DE genes were removed from the subset.

Differential gene expression data was analyzed for the pairwise comparison of the breeding line 60184 versus ‘Okeechobee’ because these genotypes have the largest difference in shelf-life phenotype the three genotypes. DE genes were separated into significantly upregulated and downregulated subsets. Gene functional enrichment for these subsets was analyzed using the STRING database^57^ for functional protein networks and the PANTHER GO Enrichment Analysis tool in the Gene Ontology Resource^58–60^. STRING identified GO enrichments, KEGG pathways with enriched protein members, and STRING local cluster networks. A subset significantly DE genes and putative SAGs was selected for preharvest analysis by RT-qPCR. The datasets generated and/or analyzed during the current study are available in the Gene Expression Omnibus (GEO) repository database, accession number GSE226302.

### Postharvest validation

Validation of candidate genes for postharvest was performed using real-time quantitative PCR (RT-qPCR). Tissue was sampled from harvested heads (*n* = 3) of the experimental line 60184 (SSL) and ‘Okeechobee’ (LSL) stored at 5 °C and 95–98% relative humidity to simulate commercial conditions and stored at 22 °C and 60–70% relative humidity to simulate accelerated senescence. Tissue samples were excised from the outer leaves of the lettuce heads at 0 day, 3 days, and 5 days after harvesting. The samples were immediately frozen in liquid nitrogen and stored at − 80 °C prior to RNA extraction^61^. Total RNA was isolated from lettuce leaves using TRIzol reagent (Invitrogen, Waltham, MA, USA) following the manufacturer’s protocol. Extracted RNA was subjected to DNase treatment using the Turbo DNA-Free Kit (Thermo Fisher Scientific, Waltham, MA, USA).

### Real-time quantitative PCR

cDNA was synthesized from 1 µg of total RNA, using the iScript cDNA Synthesis Kit (Bio-Rad, Hercules, CA, USA). Relative expression was measured between genotypes using three technical replicates of each of three biological replicates per genotype, for a total of nine replicates per genotype. Lettuce *Actin2* served as the internal reference control gene^62,63^. Total reaction volume was 20 µL with the following thermocycler conditions: 95 °C for 10 min, 45 cycles for 95 °C for 30 s, 60 °C for 30 s^64^. Relative gene expression was calculated by ∆∆C_T_ method^65^. A list of RT-qPCR primers used in this study can be found in Supplementary Table S5.

### Statistical analysis

Data for visual evaluation of shelf-life was analyzed using a nonparametric statistic method to obtain an ANOVA-Type^66,67^. The analysis was conducted in SAS (SAS v9.4) as a repeated measures approach as the data was registered over time periods (storage time). In the ANOVA, the repeated statement was “r corr” as covariance structure as indicated by^67^. The R software environment^68^ was used for statistical analyses of the leaf histological data. One-way Analysis of Variance (ANOVA) tests with post-hoc Tukey’s Honestly Significant Difference (HSD) tests were used to analyze the differences among cultivar mean ECA, lamina thickness, stomatal density, and stomatal index^7^. Four biological replicates were used per lettuce cultivar. Differences in means with a *p*-value < 0.05 were considered significant.

### IUCN policy statement

Our experimental research on lettuce plants, including the collection of plant material, complied with relevant institutional, national, and international guidelines and legislation. Plant species at risk of extinction were not used in this study.

### Supplementary Information

Supplementary Figure S1.

Supplementary Table S1.

Supplementary Table S2.

Supplementary Table S3.

Supplementary Table S4.

Supplementary Table S5.

## Data Availability

The datasets generated and/or analyzed during the current study are available in the Gene Expression Omnibus (GEO) repository database, accession number GSE226302. Token: yvajuegsjdqjdqj.
