# Supplementary figures and images for: Examining preharvest genetic and morphological factors contributing to lettuce (Lactuca sativa L.) shelf-life
Source: Sci Rep. 2024 Mar 19;14:6618. doi: 10.1038/s41598-024-55037-1 (PMC10951199; doi:10.1038/s41598-024-55037-1)

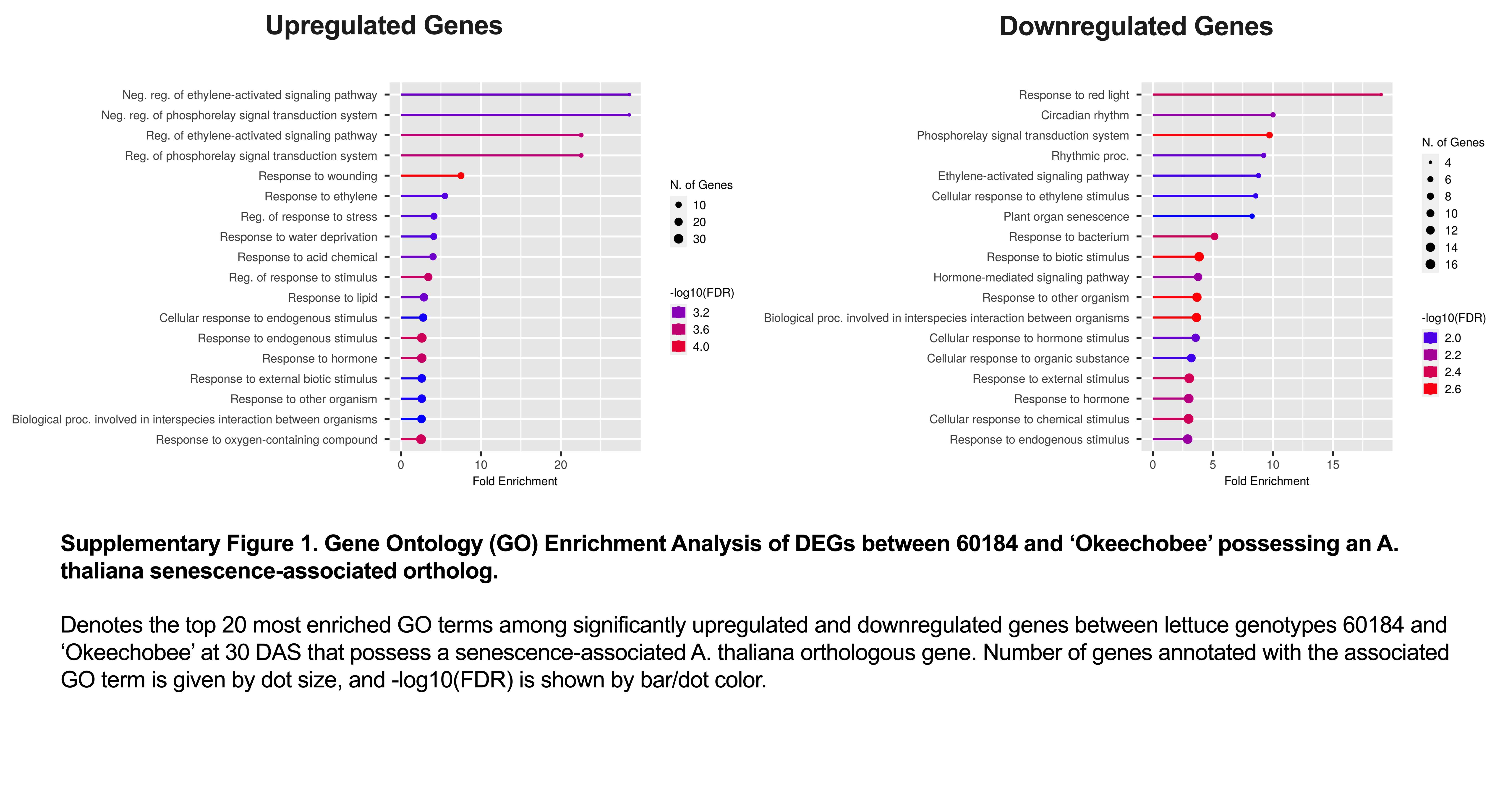

Supplement: Supplementary file 1 — Supplementary Figure S1. [file 41598_2024_55037_MOESM1_ESM.tiff]
