# Supplementary material for: Examining preharvest genetic and morphological factors contributing to lettuce (Lactuca sativa L.) shelf-life
Source: Sci Rep. 2024 Mar 19;14:6618. doi: 10.1038/s41598-024-55037-1 (PMC10951199; doi:10.1038/s41598-024-55037-1)
Supplement: Supplementary file 2 — Supplementary Table S1. [file 41598_2024_55037_MOESM2_ESM.docx]

**Supplementary Table 1. RNA-seq read alignment statistics**

| **Sample name** | **Uniquely mapped reads** | **% of reads uniquely mapped** | **% of reads mapped to multiple loci** | **% of reads unmapped** | **Mapped transcripts** |
| --- | --- | --- | --- | --- | --- |
| Okeechobee-1 | 34,918,900 | 83.77 | 7.32 | 8.89 | 25,109 |
| Okeechobee-2 | 31,124,064 | 86.75 | 3.74 | 9.49 | 24,971 |
| Okeechobee-3 | 27,436,951 | 84.89 | 6.15 | 8.95 | 25,192 |
| 60184-1 | 35,557,360 | 87.57 | 6.52 | 5.89 | 24,545 |
| 60184-2 | 33,091,107 | 88.36 | 5.25 | 6.37 | 25,029 |
| 60184-3 | 35,314,566 | 88.39 | 5.68 | 5.91 | 25,098 |
