# Supplementary material for: Examining preharvest genetic and morphological factors contributing to lettuce (Lactuca sativa L.) shelf-life
Source: Sci Rep. 2024 Mar 19;14:6618. doi: 10.1038/s41598-024-55037-1 (PMC10951199; doi:10.1038/s41598-024-55037-1)
Supplement: Supplementary file 6 — Supplementary Table S5. [file 41598_2024_55037_MOESM6_ESM.docx]

**Supplementary Table 5. Primer Sequences**

| **Application** | **Gene Name** | **Primer Name** | **Primer Sequence (5’-> 3’)** |
| --- | --- | --- | --- |
| Shelf-life determination | ORESARA1 | LSAT_ORESARA1-FP | CCAAGGGTGAATGGGTGATTAG |
| Shelf-life determination | ORESARA1 | LSAT_ORESARA1-RP | GACCCGAAGTCATGTTCATAGG |
| Shelf-life determination | ORE15 | LSAT_ORE15-FP | TTGAACCAGAGGGCACAAA |
| Shelf-life determination | ORE15 | LSAT_ORE15-RP | CCTTGCAAGATAGAGAGCAGAA |
| Preharvest RNA-seq validation | ATPase, LRR protein | 8_121540-F | CTTCCTTGTCGGTGCTATTC |
| Preharvest RNA-seq validation | ATPase, LRR protein | 8_121540-R | CCAAGCTCAATACGGGTAAG |
| Preharvest RNA-seq validation | LHCB2 | 9_104740-F | CCTTGACTACCTTGGAAACC |
| Preharvest RNA-seq validation | LHCB2 | 9_104740-R | AGACCCACCCGGATAAAT |
| Preharvest RNA-seq validation | NAC83 | 3_31741-F | GTGTTCTACCGAGGCAAAG |
| Preharvest RNA-seq validation | NAC83 | 3_31741-R | CTCAACTCTCACATCGTTCTC |
| Preharvest RNA-seq validation | LRR protein | 6_42241-F | AGTGTCCTGGAGATGAAGAA |
| Preharvest RNA-seq validation | LRR protein | 6_42241-R | CGGCGGTTGAGAAGTAAAG |
| Preharvest RNA-seq validation | ACAA1 | 5_179700-F | TCAACCTACTGCTTCATCTAATC |
| Preharvest RNA-seq validation | ACAA1 | 5_179700-R | CATCAGGAAGAGTGTCCTTAAA |
| Preharvest RNA-seq validation | K-box/MADS box family TF | 4_10400-F | AGAGAGAACCACCCAAGAA |
| Preharvest RNA-seq validation | K-box/MADS box family TF | 4_10400-R | GCATGAAGTTCCTCCTCTAAC |
| Preharvest RNA-seq validation | NAM protein | 4_1821-F | GAACCAGGAAGAAGAGAAAGAG |
| Preharvest RNA-seq validation | NAM protein | 4_1821-R | GTCTCACGGCAACCATAAA |
| Preharvest RNA-seq validation | WRKY domain protein | 9_39381-F | GCCAAGCGACAGGATAAA |
| **Application** | **Gene Name** | **Primer Name** | **Primer Sequence (5’-> 3’)** |
| Preharvest RNA-seq validation | WRKY domain protein | 9_39381-R | GCAAGAGCCCAAGATTAAGA |
| Preharvest RNA-seq validation | GLK1-like protein | 4_65921-F | CCATCTCCTTCACCATCTTG |
| Preharvest RNA-seq validation | GLK1-like protein | 4_65921-R | CCAATGCCGGAGTCTATTT |
| Postharvest RNA-seq validation | GA 2-oxidase 8 | 1_122260-F | CCAAAGCCTCGCAAGAATG |
| Postharvest RNA-seq validation | GA 2-oxidase 8 | 1_122260-R | CCTCACCTGTTCACATCTCATC |
| Postharvest RNA-seq validation | K-box/MADS-box | 4_10400-F | TCTGTGATGTTGATGTCGCC |
| Postharvest RNA-seq validation | K-box/MADS-box | 4_10400-R | CTTGGGTGGTTCTCTCTTCTG |
| Postharvest RNA-seq validation | Pectate lyase | PL_3_qPCR_F | GCGTGACTATGATGATGGACTT |
| Postharvest RNA-seq validation | Pectate lyase | PL_3_qPCR_R | GAGATGGATCCGCTCCAATAAG |
| Postharvest RNA-seq validation | Expansin A9/A4 | EXP_2_qPCR_F | CTCGCCCTCATTTCGATCTC |
| Postharvest RNA-seq validation | Expansin A9/A4 | EXP_2_qPCR_R | ACCTTATCCCACCTTGTTTCC |
